# *Bacteroides fragilis* requires the ferrous‐iron transporter FeoAB and the CobN‐like proteins BtuS1 and BtuS2 for assimilation of iron released from heme

**DOI:** 10.1002/mbo3.669

**Published:** 2018-06-21

**Authors:** Edson R. Rocha, Hector A. Bergonia, Svetlana Gerdes, Charles Jeffrey Smith

**Affiliations:** ^1^ Department of Microbiology and Immunology Brody School of Medicine Greenville North Carolina; ^2^ Iron and Heme Core Division of Hematology University of Utah School of Medicine Salt Lake City Utah; ^3^ Fellowship for Interpretation of Genomes Burr Ridge Illinois

**Keywords:** anaerobes, anaerobic bacteria, Bacteroides, chelatase, dechelatase, demetallase, heme, iron

## Abstract

The intestinal commensal and opportunistic anaerobic pathogen *Bacteroides fragilis* has an essential requirement for both heme and free iron to support growth in extraintestinal infections. In the absence of free iron, *B. fragilis* can utilize heme as the sole source of iron. However, the mechanisms to remove iron from heme are not completely understood. In this study, we show that the inner membrane ferrous iron transporter *∆feoAB* mutant strain is no longer able to grow with heme as the sole source of iron. Genetic complementation with the *feoAB* gene operon completely restored growth. Our data indicate that iron is removed from heme in the periplasmic space, and the released iron is transported by the FeoAB system. Interestingly, when *B. fragilis* utilizes iron from heme, it releases heme‐derived porphyrins by a dechelatase activity which is upregulated under low iron conditions. This is supported by the findings showing that formation of heme‐derived porphyrins in the *∆feoAB* mutant and the parent strain increased 30‐fold and fivefold (respectively) under low iron conditions compared to iron replete conditions. Moreover, the *btuS1 btuS2* double‐mutant strain (lacking the predicted periplasmic, membrane anchored CobN‐like proteins) also showed growth defect with heme as the sole source of iron, suggesting that BtuS1 and BtuS2 are involved in heme‐iron assimilation. Though the dechelatase mechanism remains uncharacterized, assays performed in bacterial crude extracts show that BtuS1 and BtuS2 affect the regulation of the dechelatase‐specific activities in an iron‐dependent manner. These findings suggest that the mechanism to extract iron from heme in *Bacteroides* requires a group of proteins, which spans the periplasmic space to make iron available for cellular functions.

## 1 INTRODUCTION

*Bacteroides fragilis* is among the smallest components of the commensal *Bacteroides* species found in the human gut ranging from <0.5% to 4% of the total microflora (Holdeman, Good, & Moore, 1976; Kraal, Abubucker, Kota, Fischbach, & Mitreva, 2014). However, *B. fragilis* emerges as the most prevalent anaerobic organism in human infections (Finegold & George, 1989; Mazuski & Solomkin, 2009; McClean, Sheehan, & Harding, 1994; Park, Choi, Yong, Lee, & Kim, 2009; Smith, Rocha, & Paster, 2006). *B. fragilis* opportunistic infections occur as a consequence of a disruption in the integrity of the intestinal mucosa wall. Most of the bacteria leaked into the peritoneal cavity from the lumen is rapidly cleared by host defenses, but the anaerobe *B. fragilis* and facultative bacteria such as *Escherichia coli* often escape clearance and are the predominant organisms found in resulting abscess infections. As consequence, the majority of intraabdominal infections are of polymicrobial nature (Edmiston, Krepel, Seabrook, & Jochimsen, 2002; Johnson, Baldessarre, & Levison, 1997). *Bacteroides fragilis* accounts for 50%–90% of all anaerobes isolated from infections of which peritonitis, intraabdominal abscesses, and bacteremia are the predominant morbidities. In the event of a rupture in the abscess wall, *B. fragilis* may gain access to the bloodstream often leading to septic shock and systemic organ failure. *Bacteroides fragilis* is a deadly pathogen as it accounts for 5% of all positive blood cultures with mortality rates of 16%–45%. Despite the high incidence of *B. fragilis* in intraabdominal infections, the full range of virulence factors that allow it to arise as a predominant opportunistic anaerobic pathogen remain to be understood (Blairon et al., 2006; Brook, 1989, 2010; Brook & Frazier, 2000; Cheng et al., 2009; Finegold & George, 1989; Mazuski & Solomkin, 2009; Nguyen et al., 2000; Park et al., 2009; Salonen, Eerola, & Meurman, 1998; Wilson & Limaye, 2004; Yoshino et al., 2012).

A factor that plays an important role in survival of *B. fragilis* in extraintestinal infection is its ability to acquire heme and inorganic iron from host tissues (Otto, van Dooren, Dozois, Luirink, & Oudega, 2002; Veeranagouda et al., 2014). Moreover, when *B. fragilis* is coinfected with *E. coli* strains producing hemoglobin protease during experimental polymicrobial infection, *E. coli* facilitates *B. fragilis* to utilize iron from heme to overcome host iron restriction mechanisms (Otto et al., 2002). *Bacteroides fragilis* as well as all other *Bacteroides* spp. have an essential requirement for heme and inorganic iron, and growth is stimulated by heme in a dose‐dependent manner. In the absence of exogenous inorganic iron, *B. fragilis* is able to utilize heme as the source of iron in vitro (Chen & Wolin, 1981; Fuller & Caldwell, 1982; Rocha, de Uzeda, & Brock, 1991; Rocha & Smith, 2010; Sperry, Appleman, & Wilkins, 1977; Varel & Bryant, 1974). The growth stimulation of *Bacteroides* by heme was shown to be due to activation of the fumarate reductase complex involved in the reduction of fumarate to succinate for energy generation during glucose fermentation (Baughn & Malamy, 2003; Caldwell, White, Bryant, & Doetsch, 1965; Chen & Wolin, 1981; Harris & Reddy, 1977; Macy, Probst, & Gottschalk, 1975; Rocha & Smith, 2010; Sperry et al., 1977).

The heme‐dependence of *Bacteroides* spp. is due to their inability to synthesize precursors of the macrocycle tetrapyrrole ring protoporphyrin IX (PpIX). This is a distinctive characteristic of host‐associated Bacteroidetes in the genera *Bacteroides*,* Prevotella,* and *Porphyromonas* which are unable to synthesize their own PpIX due to the lack of most genes required for the formation of the tetrapyrrole macrocycle, though they can synthesize heme in vitro if PpIX and inorganic iron are supplied (Olczak, Simpson, Liu, & Genco, 2005; Rocha & Smith, 2010). Interestingly, this is a common characteristic of many bacterial species that colonize the lower intestinal tract of humans and other animals such as *Bifidobacterium*,* Enterococcus*,* Lactococcus*,* Clostridium,* and euryarchaeota (Rocha & Smith, 2010). In contrast, free living Bacteroidetes such as *Flavobacterium*,* Cytophaga,* and *Salinibacter* contain a complete heme biosynthesis pathway (Rocha & Smith, 2010). The Bacteroidetes can synthesize heme in vitro if PpIX and inorganic iron are provided, however, *Bacteroides* and *Prevotella*, do not contain homologs to the classical ferrochelatase HemH (PpfC) (Rocha & Smith, 2010; for review on heme biosynthesis in prokaryotes, see Dailey et al., 2017). This suggests that they utilize a novel mechanism to incorporate iron into PpIX for the synthesis of heme (Rocha & Smith, 2010).

Another aspect of heme metabolism in *B. fragilis* that has received little attention is its ability to dechelate divalent metal‐porphyrins. Support for the presence of a dechelatase mechanism in *B. fragilis* is provided by several lines of evidence demonstrating that *Bacteroides*/*Prevotella* are versatile in the removal of nonferrous divalent metal‐bound porphyrins and side chain‐modified porphyrins (such as Mn‐PpIX, Mg‐PpIX, Mn‐mesoporphyrin IX, Mg‐mesoporphyrin IX, Mn‐deuteroporphyrin IX, or Mg‐deuteroporphyrin IX). After dechelation, ferrous iron is inserted into the porphyrin free metal pocket through an unidentified ferrochelatase to form heme, mesoheme or deuteroheme, respectively. These activities seem to be relevant for cellular functions because heme itself or side chain‐modified heme (mesoheme and deuteroheme) can be incorporated into the cytochrome *b*‐type of the fumarate reductase complex and they are equally functional physiologically (Caldwell et al., 1965; Fuller & Caldwell, 1982; Gardner, Fuller, & Caldwell, 1983; McCall & Caldwell, 1977).

This resourceful tetrapyrrole utilization appears to be important for the metabolism of heme in extraintestinal infections as well as during intestinal tract colonization. The formation of heme‐derived porphyrin in abscess pus is associated with the presence of anaerobic bacteria and the porphyrin pattern is remarkably similar to heme‐derived porphyrin in the intestinal tract (Brazier, 1990). The exogenous source of heme in abscesses may be provided by extravasated red blood cells and polymorphnuclear leukocytes (Brazier, 1990). In the GI tract, the unabsorbed heme that reaches the lower intestinal tract together with nonpathological sources of luminal heme (including physiological epithelial shedding of cells and microbleeding) are converted by bacteria to a range of heme‐derived porphyrins (Beukeveld et al., 1987; Rose et al., 1989; Young, Rose, & St John, 1989; Young, Rose, St John, & Blake, 1990). The appearance and fluctuation of deuteroheme, mesoheme, and pemptoheme contents and conversion of heme‐derived porphyrins into deutero‐ meso‐, and pempto‐porphyrins in feces of healthy human subjects depends entirely and exclusively on the anaerobic bacterial flora (Beukeveld et al., 1987; Rocha & Smith, 2010; Rose et al., 1989; Young et al., 1989, 1990). The aerobic and facultative anaerobic microbiota component of the intestinal tract plays no or a negligible role in the production of heme‐derived porphyrins in the gut (Beukeveld et al., 1987). However, there is a paucity of information on the mechanisms of heme‐iron assimilation and its contribution to the formation of heme‐derived porphyrins in *B. fragilis*.

Though progress has been made to establish that heme is a major source of iron for *Bacteroides* both in vitro and in vivo, the mechanisms involved in heme‐iron acquisition have not been fully characterized. In addition, there is a paucity of information regarding the identification of the lower intestinal anaerobic bacteria species involved in heme dechelation and porphyrin side‐chain modifications. In this study we show that when *B. fragilis* is grown on heme as the sole source of iron, the ability to remove iron from heme releasing free PpIX is regulated by iron availability. We provide evidence that iron is removed from heme extracytoplasmically and assimilation of iron released from heme to stimulate bacterial growth is dependent on the presence of the inner‐membrane ferrous iron transporter system, FeoAB. Moreover, this study shows that BtuS, a member of the CobN‐like family of proteins (Rodionov, Vitreschak, Mironov, & Gelfand, 2003), is involved in heme‐iron assimilation. Therefore, this study demonstrates that heme and iron metabolism in *B. fragilis* differs from the classical aerobic and facultative anaerobic bacterial systems.

## 2 MATERIALS AND METHODS

### 2.1 Bacterial strains and growth conditions

*Bacteroides fragilis* strains used in this study are shown in Table 1. Strains were routinely grown anaerobically in brain heart infusion medium (BHIS) containing l‐cysteine (1 g/L) and supplemented with hemin (5 mg/L) or otherwise stated in the text. After autoclaving, 20 ml of 10% NaHCO_3_ per liter was added into the BHIS medium. For some experiments hemin was replaced with PpIX as delineated in the text. In an aqueous solution, it is not always possible to define completely the modified forms of heme (strictly ferrous iron‐protoporphyrin IX) macrocycle due to the variation in the iron valence and the salts formed (see Smith, 1990 for review). In addition, heme in an aqueous solution for an extended time can lead to formation of oxo‐μ‐dimers of heme (Smith, 1990). Thus, we hereafter use the term heme to refer to an iron–protoporphyrin IX complex without specifically referring to its structural form or valence. Twenty μg/ml rifamycin, 100 μg gentamicin/ml, 5 μg tetracycline/ml, and 10 μg erythromycin/ml were added to the media when required. Media were supplemented with the ferrous iron chelator bathophenanthroline disulfonic acid (BPS), which does not enter the cell, to obtain iron‐limiting conditions as previously described (Rocha & Krykunivsky, 2017). Addition of ferrous iron sulfate or ammonium ferrous iron sulfate was used to obtain iron‐replete conditions. In some experiments, defined medium (DM) was used as described previously (Rocha & Krykunivsky, 2017) to determine the stimulatory effect of heme and PpIX on the bacterial growth rate.

**Table 1:** *Bacteroides* strains and plasmids used in this study

	Relevant genotype	References
Strains		
*B. fragilis* 638R	clinical isolate, Rif^R^	Privitera, Dublanchet, & Sebald (1979)
*B. fragilis* BER‐2	638R *∆furA::cfxA*, Rif^R^ Cfx^R^	Robertson et al. (2006)
*B. fragilis* BER‐51	638R *∆feoAB::tetQ*, Rif^R^ Tet^R^	Veeranagouda et al. (2014)
*B. fragilis* BER‐53	638R *∆feoAB::tetQ, ∆furA::cfxA*, Rif^R^ Tet^R^ Cfx^R^	This study
*B. fragilis* BER‐125	638R *∆feoAB::tetQ*,* feoAB* ^*+*^ Tet^R^ Erm^R^	Veeranagouda et al. (2014)
*B. fragilis* BER‐107	638R *btuS1*::pFD::516 Rif^R^ Erm^R^	This study
*B. fragilis* BER‐109	638R *btuS2*::pFD516 Rif^R^ Erm^R^	This study
*B. fragilis* BER‐111	638R *btuS1*::pFD516 *btuS2*::pYT102 Rif^R^ Erm^R^ Tet^R^	This study
*B. fragilis* BER‐114	BER‐111 pER‐179 Rif^R^ Erm^R^ Tet^R^ Cfx^R^	This study
*B. fragilis* BER‐115	BER‐111 pER‐180 Rif^R^ Erm^R^ Tet^R^ Cfx^R^	This study
Plasmids		
pYT102	*Bacteroides* suicide vector. (Cm^R^), Tet^R^	Baughn & Malamy (2002)
pFD340	*Bacteroides* expression shuttle vector. (Amp^R^), Erm^R^	Smith et al. (1992)
pFD516	*Bacteroides* suicide vector. (Sp^R^), Erm^R^	Smith et al. (1995)
pER‐167	A 528 nt internal N‐terminus DNA fragment of *butS2* gene was cloned into the suicide vector pFD516	This study
pER‐168	An approximately 694 nt internal N‐terminus DNA fragment of *butS1* gene was cloned into the suicide vector pFD516	This study
pER‐173	An approximately 1,200 nt internal N‐terminus DNA fragment of *butS1* gene was cloned into the suicide vector pYT102	This study
pER‐178	An approximately 2,400 nt BamHI/EcoRI DNA fragment from pFD340 was deleted and replaced with an approximately 2.4 kb *cfxA* gene into the BamHI/EcoRI sites. (Amp^R^) Cfx^R^	Rocha & Krykunivsky (2017)
pER‐179	A 4,094 nt promoterless *btuS1* gene was cloned into the BamHI site of pER‐178 in the same orientation of IS4315	This study
pER‐180	A4,490 nt promoterless *btuS2* gene was cloned into the BamHI site of pER‐178 in the same orientation of IS4315	This study

*Note*. Erm^R^: erythromycin resistance; Cfx^R^: cefoxitine resistance; Rif^R^: rifamycin resistance; Tet^R^: tetracycline resistance; Cm^R^: chloramphenicol resistance. Amp^R^: ampicillin resistance; Sp^R^: spectinomycin resistance. Parenthesis indicate antibiotic resistance in *Escherichia coli*.

### 2.2 Construction of *∆feoAB ∆furA* double‐mutant

To construct a *∆feoAB ∆furA* double mutant strain, the plasmid pER‐66 containing the *∆feoAB::tetQ* deletion construct in *E. coli* DH10B (Veeranagouda et al., 2014) was mobilized into the *B. fragilis ∆furA::cfxA* (BER‐2) strain (Robertson, Smith, Gough, & Rocha, 2006) by aerobic triparental mating as described previously (Rocha & Smith, 2004). Transconjugants were selected on BHIS plates containing 20 μg rifamycin/ml, 100 μg gentamicin/ml, 25 μg cefoxitin/ml, and 5 μg tetracycline/ml. Determination of sensitivity to either tetracycline or erythromycin was carried out to identify recombinants that were tetracycline resistant and erythromycin sensitive. PCR amplification analysis was used to confirm the double‐crossover genetic allele exchange of pER‐66 into the *B. fragilis* BER‐2 chromosome. The new transconjugant BER‐53 containing the *∆furA::cfxA ∆feoAB::tetQ* genotype was selected for further studies.

### 2.3 Construction of *btuS1* and *btuS2* insertional mutants

A 528 nt fragment was amplified from the N‐terminus region of *B. fragilis* 638R_2505 (*btuS1*) gene using primers GAGGCGGA**T**C**C**TGCCGCATCG, and CGAAT**G**A**G**CTCCAAGTCTTCC containing modified nucleotides (bold font) and restriction sites (underlined) for BamHI and SstI, respectively. The amplified fragment was cloned into the BamHI/SstI sites of the suicide vector pFD516 (Smith, Rollins, & Parker, 1995). The new construct pER‐167 was mobilized from *E. coli* DH10B into *B. fragilis* 638R by aerobic triparental filter mating protocols as mentioned above. The transconjugants were selected on BHIS agar plates containing 20 μg rifampicin/ml, 100 μg gentamicin/ml, and 10 μg erythromycin/ml. The *B. fragilis btuS1*::pFD516 insertion mutant, strain BER‐107 was subjected to PCR analysis to confirm the single cross‐over disruption of the target gene.

A 694 nt fragment was amplified from the N‐terminus region of *B. fragilis* 638R_2718 (*btuS2*) gene using primers GTTGTGTGG**A**T**C**CGGCAATACTCG, and CGGACGA**G**CTCCATGAAACGG containing modified nucleotides (bold font) and restriction sites (underlined) for BamHI and SstI, respectively. The amplified fragment was cloned into the BamHI/SstI sites of the suicide vector pFD516 and the new construct pER‐168 was mobilized from *E. coli* DH10B into *B. fragilis* 638R by aerobic triparental filter as above. The transconjugants were selected on BHIS agar plates containing 20 μg rifampicin/ml, 100 μg gentamicin/ml, and 10 μg erythromycin/ml. The *B. fragilis btuS2*::pFD516 insertion mutant, strain BER‐109 was subjected to PCR analysis to confirm the single cross‐over disruption of the target gene.

### 2.4 Construction of *btuS1 btuS2* double insertional mutant

A 1,174 nt fragment was amplified from the N‐terminus region of *B. fragilis* 638R_2718 (*btuS2*) gene using primers GTTGTGTGG**A**T**C**CGGCAATACTCG, and CTCTCCGGAAG**C**TTTTCTACCCGG containing modified nucleotides (bold font) and restriction sites (underlined) for BamHI and HindIII, respectively. The amplified fragment was cloned into the BamHI/HindIII sites of the suicide vector pYT102 (Baughn & Malamy, 2002). The new construct pER‐173 was mobilized from *E. coli* DH10B into BER‐107 strain by aerobic triparental filter mating protocols as described above. The transconjugants were selected on BHIS agar plates containing 20 μg rifampicin/ml, 100 μg gentamicin/ml, 10 μg erythromycin/ml and 5 μg tetracycline/ml. The *B. fragilis btuS1*::pFD516 *btuS2*::pYT102 double insertion mutant, strain BER‐111 was subjected to PCR analysis to confirm the single cross‐over disruption of the target gene.

For genetic complementation of BER‐111 strain with *btuS1* gene, a 4,094 nt promoterless DNA fragment of the BF638R_2505 gene locus containing 45 nt upstream the ATG codon and 85 nt downstream from the stop codon was PCR amplified using primers GGAGTTG**G**A**TC**CGGAGTGAG and CAACCAG**GA**TCCCCATCGCG, modified to include restriction sites as described above. This fragment was then cloned into the BamHI site of pER‐178 (Rocha & Krykunivsky, 2017). Expression of the *btuS1* gene is driven by the constitutive IS4351 promoter from the original expression vector pFD340 (Table 1, Smith, Rogers, & McKee, 1992). The new construct, pER‐179 was conjugated into BER‐111 by triparental mating to obtain BER‐114.

For genetic complementation of BER‐111 strain with *btuS2* gene, a 4,490 nt promoterless DNA fragment of the BF638R_2718 gene locus containing 50 nt upstream the ATG codon and 94 nt downstream from the stop codon was PCR amplified using primers GATATCTC**G**GATC**C**TCCGGC and GTGGTTA**G**GAT**C**CCGACTGC, modified to include restriction sites as described above. This fragment was then cloned into the BamHI site of pER‐180 (Rocha & Krykunivsky, 2017). Expression of the *btuS2* gene is driven by the constitutive IS4351 promoter from the original expression vector pFD340 (Table 1, Smith et al., 1992). The new construct, pER‐179 was conjugated into BER‐111 by triparental mating to obtain BER‐115.

### 2.5 Determination of total heme and total porphyrin in whole cell dry extract

We used a modified spectrophotometric method described by Kufner, Schelegel, and Jager (2005) to extract and quantify total porphyrin and heme in dried bacterial pellets. Bacteria were grown on BHIS plates containing 100 μg/ml heme and supplemented with either 100 μM ferrous ammonium sulfate or 500 μM BPS for 48–72 hr. Bacteria were washed from the plate surface with 145 mM NaCl and centrifuged at 12,000*g* for 10 mins at 4°C. The pellets were washed once with 145 mM NaCl and transferred to a preweighed 12 ml Falcon round‐bottom tubes and centrifuged at 12,000*g* for 10 mins at 4°C. The cell pellets were lysed by the addition of 200 μl chloroform and dried at 65°C. To the dried pellets, 550 μl releasing solution [hydroxyquinone 10 g/L in formic acid/water 75:25 v/v] was added, vortexed and incubated overnight at 4°C. Then 2 ml diethyl ether was added, and samples were vortexed and left at 4°C for 1–2 hr with frequent vortexing. The suspensions were centrifuged at 5,000*g* for 5 min to remove debris and insoluble material. Pellets were discarded and 1 ml of 2.5 mol/L HCl was added to the supernatants, which were mixed and kept at 4°C overnight. After centrifugation, the upper ether layer was removed for heme determination. To the lower acid layer, 2 ml of diisopropyl ether and 0.5 ml ammonium formate solution (500 g/L) was added. The mixtures were vortexed continuously for a few minutes and centrifuged. The lower buffer layer was discarded. To the ether upper layer, 1 ml of 2.5 mol/L HCl was added and samples were vortexed continuously for a few minutes. After centrifugation, the upper ether layer was discarded. The lower acid layers were used to measure porphyrin concentration. The light protective solution (hydroxyquinone 20 mg/L in 2.5 mol/L HCl) was prepare before use and used as measuring and blank solution in the determination of porphyrin content in the lower acid layer. Total protoporphyrin IX was calculated using millimolar extinction at 409 nm (m*E*_409_) = 295.6 (Kufner et al., 2005) and normalized to nmol/mg dry weight. Total heme content was determined by pyridine‐NaOH as described previously (Rocha & Smith, 1995). Bovine hemin (Sigma) was used as the standard.

### 2.6 Reverse phase HPLC analysis of porphyrin extract

Acid extract fractions obtained as described above were filtered in a 0.2 μm nylon filter membrane before injecting samples with an autosampler attached to the Shimadzu HPLC SIL‐20AHT system with dual spectrofluorometric detector RF‐20A (Shimadzu Scientific Instruments, Columbia, MD). A 250 × 4.6 mm ID column packed with SynChropak RPP‐100 (SynChrom, Inc) was used with a 50 × 4.6 mm ID guard precolumn containing the same RPP‐100 material (100 Å pore size 5 μm particles) (Eprogen Inc., Darien, IL). To detect porphyrins the spectrofluorometer was set up with the following conditions: excitation 408 nm (15 nm bandwidths) and emission 620 nm (15 nm bandwidths). To separate the porphyrins using the C18 reverse phase column, we used eluent A (1,000 ml double‐deionized, 0.5 ml dimethylpyridine, 0.8 ml phosphoric acid (85%), 210 ml acetonitrile HPLC grade, 140 ml acetone), and eluent B (600 ml acetonitrile, 300 ml methanol, 100 ml acetone) as described by Beukeveld et al. (1987). The porphyrin separation solvent gradient parameters were conditioned as follows: 0% B, increasing to 95% B in 19 min, then 97% B for 5 min. This was followed by a return to 0% B in 3 min and a run for 13 min before injection of the new sample according to Beukeveld et al. (1987). The liquid flow rate was 1.4 ml/min. The porphyrin acids chromatographic marker kit containing mesoporphyrin IX, 4 carboxyl porphyrin [coproporphyrin I], 5 carboxyl porphyrin, 6 carboxyl porphyrin, 7 carboxyl porphyrin, and 8 carboxyl porphyrin [uroporphyrinogen I] was purchased from Frontier Scientific, Logan, UT. Deuteroporphyrin IX dihydrochloride, mesoporphyrin IX dihydrochloride, and pemptoporphyrin were purchased from Frontier Scientific. Protoporphyrin IX disodium salt was purchased from Sigma‐Aldrich Co., St. Louis, MO. The standard markers were dissolved in 2.7 mol/L HCl to obtain 2 nmol/ml solutions.

### 2.7 Determination of the forward and reverse chelatase activity in whole cell crude extracts

Bacteria were grown on 100 ml BHIS broth containing 10 μg heme/ml with 100 μM ammonium ferrous sulfate or 500 μM BPS. Bacteria were grown for 24–48 hr on iron‐replete media. For growth in iron‐limiting conditions, the cultures were incubated for 72–96 hr. BHIS broth containing 10 μg PpIX/ml instead of heme was also used. Bacterial cultures were centrifuged at 12,000*g* for 10 mins at 4°C. The pellets were washed once with phosphate buffered saline (PBS) [10 mM Na_2_HPO_4_, 1.7 mM KH_2_PO_4_, 145 mM NaCl, 2 mM KCl, pH 7.4] and centrifuged as above. The pellets were frozen at −70°C and submitted to the Iron and Heme Core facility, Department of Internal Medicine, Division of Hematology, University of Utah Health Sciences, Salt Lake City, UT, to assay for ferrochelatase and dechelatase specific activities in bacterial crude extracts. The ferrochelatase assay described on the Iron and Heme core website (http://cihd.cores.utah.edu/ironheme/#1465838003097-f4fffc70-f824) is a modification of the method described by Rossi, Costin, & Garcia‐Webb (1988). The dechelatase (reverse chelatase) assay was adapted from the methods described by Chau, Ishigaki, Kataoka, & Taketani (2010) and Taketani et al. (2007). Briefly, approximately 1 mg total protein in 50 μl of bacterial crude extract was mixed with 50 μL of assay reagent containing 200 μM hemin‐imidazole, 4 mM ascorbic acid in 10 mM potassium phosphate buffer pH 5.5 under argon atmosphere. The enzyme assay was incubated at 45°C for 30 min. After the addition of 400 μL 50% v/v acetone in ethanol, the samples were spun at 13,500 g for 10 min. The supernatant was analyzed by UPLC (ultra‐performance liquid chromatography) for protoporphyrin IX fluorescence and quantification as described on the Iron and Heme core website (http://cihd.cores.utah.edu/ironheme/#1467136333172-56f37fbb-2816). Bacterial crude extract samples were heated for 10 min in a boiling water bath and used as blank controls.

## 3 RESULTS

### 3.1 The role of *feoAB* in *B. fragilis* growth with heme as the sole source of iron

**Figure 1:**
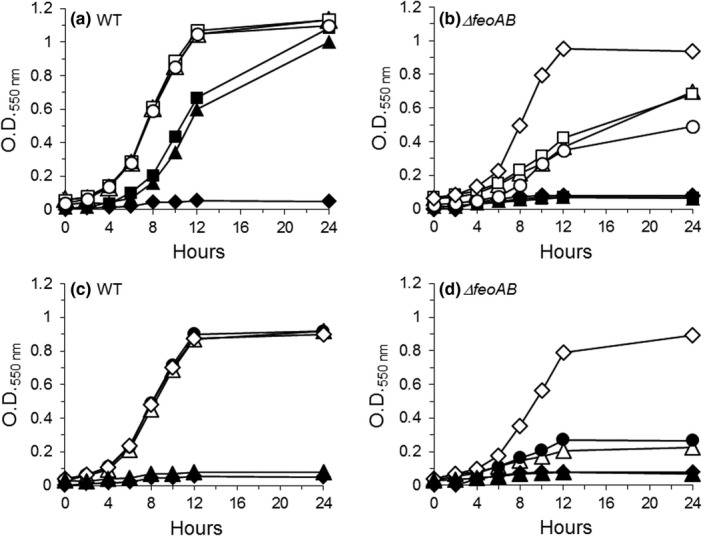
Growth of *Bacteroides fragilis* 638R (WT) and *∆feoAB* mutant strains in defined medium containing heme (He) (a and b) or protoporphyrin IX (PpIX) (c and d). Ammonium ferrous sulfate (Fe) or bathophenanthroline disulfonic acid (BPS) were added to obtain iron replete and iron‐limiting conditions, respectively. The concentrations of each supplement added into the culture media are described in the symbols legend below. (a and b) ♦ No addition; △ 5 μg He/ml; □ 10 μg He/ml; ▲ 5 μg He/ml + 400 μM BPS; ■ 10 μg He/ml + 400 μM BPS; ○ 10 μg He/ml + 2 μM Fe; ♢ 10 μg He/ml + 100 μM Fe (only shown for *∆feoAB* strain in b). (c and d) ♦ No addition; △ 5 μg PpIX/ml; ▲ 5 μg PpIX/ml + 400 μM BPS; ● 5 μg PpIX/ml + 2 μM Fe; ♦ 5 μg PpIX/ml + 100 μM Fe. Data presented are the average of two determinations in duplicate

When *B. fragilis* strains were grown in DM supplemented with heme in the presence of the ferrous iron chelator BPS, the wild‐type parent strain was able to grow in the presence of heme as the only source of iron. In contrast, the growth of the ∆*feoAB* strain was abolished indicating that the ∆*feoAB* strain could no longer obtain essential iron from heme (Figure 1a,b). Partial growth of the ∆*feoAB* occurred in the presence of 5 μg and 10 μg heme/ml compared to maximum growth rate observed for the parent stain in media where residual iron was not chelated (Figure 1a,b). When PpIX replaced heme as the tetrapyrrole macrocycle, neither the parent nor the ∆*feoAB* mutant strains were able to grow in media that was iron‐limited by the addition of BPS (Figure 1c,d). In the absence of BPS, the parent but not the *∆feoAB* strain, grew in basal medium containing PpIX without added iron, indicating that residual iron was sufficient to support the growth of the parent strain in the presence of PpIX (Figure 1c,d). The addition of 2 μM FeSO_4_ did not significantly stimulate the growth of the *∆feoAB* strain in the presence of either heme or PpIX, but addition of 100 μM FeSO_4_ restored growth to wild‐type levels (Figure 1b,d). No growth of either strain occurred in the absence of added heme or PpIX (Figure 1a,b,c,d). This indicates that in the absence of heme, exogenous PpIX and iron are essential nutrients to support growth. Taken together, these findings indicate that *B. fragilis* can utilize inorganic iron in the presence of heme or PpIX but in the absence of inorganic iron, growth can only be supported if heme is present. Although *B. fragilis* does not possess a known HemH (PpfC) homolog (Rocha & Smith, 2010), these experiments indicate that *B. fragilis* can synthesize essential heme if PpIX and iron are provided. Moreover, these findings suggest that in the absence of free iron, iron is removed from heme in the periplasmic space since the inner membrane transport system FeoAB is required to mobilize ferrous iron from the periplasmic space across the inner‐membrane into the cytoplasm. It is unlikely that iron was removed from heme outside the cell before reaching the periplasm because extracellular free iron would be readily available for chelation by BPS.

**Figure 2:**
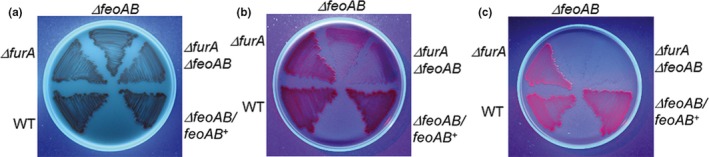
Growth of *Bacteroides fragilis* strains on brain heart infusion (BHIS) media containing 100 μg/ml hemin. Plates were supplemented with (a) 100 μM FeSO_4_. (b) 300 μM bathophenanthroline disulfonic acid (BPS) and (c) 1 mM BPS. Bacteria were grown in an anaerobic chamber at 37°C for 7 days. Plates were illuminated with a 365 nm UV‐long wave lamp (UVP; model UVLS‐28, Upland, CA) and pictures were taken with an Olympus Camedia C‐4000 digital camera. WT:* B. fragilis* 638R wild type. Strain designations are depicted on each panel

During the course of this investigation it was found that *B. fragilis* cultures grown in the presence of heme and BPS, but not under iron‐replete conditions, produced porphyrin‐like pigment when exposed to longwave‐UV light (Supporting Information Figure S1). This observation was further analyzed by culturing strains on BHIS plates containing high amount of heme at 100 μg/ml, supplemented with either 100 μM ammonium ferrous sulfate, 300 μM BPS, or 1 mM BPS. After 7 days of incubation, the plates were exposed to longwave UV light at 365 nm (Figure 2). There was no inhibition of growth or of fluorescent porphyrin production by the parent, *∆furA*,* ∆feoAB*,* ∆feoAB ∆furA,* and *∆feoAB/feoAB* ^*+*^ strains on iron‐replete culture plates (Figure 2a). In contrast, under iron‐limiting conditions, porphyrin fluorescence was clearly seen in the parent, *∆furA* and *∆feoAB/feoAB* ^*+*^ strains (Figure 2b,c). However, the growth of the *∆feoAB* and *∆feoAB ∆furA* double‐mutant strains were inhibited under iron‐limiting conditions, confirming that the formation of heme‐derived porphyrins was directly linked to heme as a source of iron. Genetic complementation of the *∆feoAB* mutation with *feoAB* gene completely restored growth when heme was the sole source of iron. Therefore, this confirms that iron is likely being removed from heme in the periplasmic space since the FeoAB system is required for its assimilation.

### 3.2 Determination of total heme and heme‐derived porphyrins in whole cell extracts

**Figure 3:**
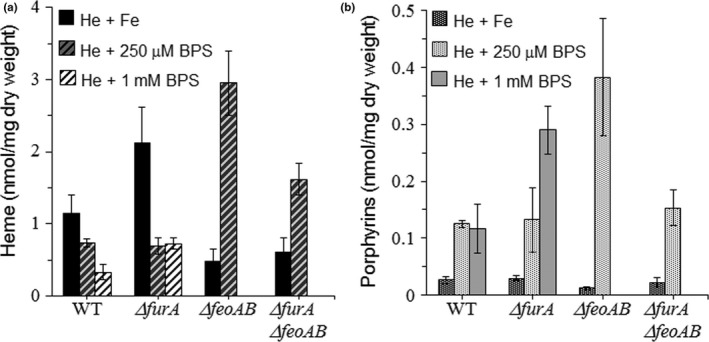
Total heme (a) and total porphyrin (b) determination in dried whole cell extracts of *Bacteroides fragilis* strains grown in brain heart infusion (BHIS) media containing 100 μg/ml hemin and supplemented with 100 μM (NH_4_)_2_Fe(SO_4_)_2_ (Fe), 250 μM bathophenanthroline disulfonic acid (BPS) or 1 mM BPS. Extraction of heme (He) and protoporphyrins (Pp) from dried cells was performed by organic/acid aqueous phase separations as described in the materials and methods section. Growth of the *∆feoAB* and *∆furA ∆feoAB* strains was inhibited by 1 mM BPS. The data presented are the mean of five independent experiments. Standard deviation bars represent deviance (±) of the mean

When the 638R parent and *∆furA* mutant strains were grown in medium containing 100 μg/ml heme and 100 μM ammonium ferrous sulfate, the total cellular heme amount was found to be approximately 1.2 nmol/mg and 2.2 nmol/mg of dry weight, respectively. In the presence of 1 mM BPS, the amount of heme decreased approximately fourfold, to 0.3 nmol/mg and 0.72 nmol/mg of dry weight, respectively (Figure 3a). In contrast, the amount of heme in the *∆feoAB* and *∆feoAB ∆furA* mutant strains increased to 2.9 and 1.62 nmol/mg of dried weight in the presence of 250 μM BPS compared to 0.48 and 0.61 nmol/mg of dried weight under iron replete conditions, respectively. No growth of the *∆feoAB* and *∆feoAB ∆furA* strains occurred in media containing 1 mM BPS (Figure 3a,b). The amount of total porphyrins found in the parent and *∆furA* strains was higher approximately fivefold and 10‐fold respectively, in the presence of 1 mM BPS (0.12 and 0.30 nmol/mg of dried weight) compared to the amount under iron‐replete conditions (0.026 and 0.029 nmol/mg of dried weight) (Figure 3b). In the *∆feoAB* strain, there was approximately a 30‐fold increase in the amount of porphyrins under iron restricted conditions (0.38 nmol/mg of dried weight) compared to iron‐replete conditions (0.012 nmol/mg of dried weight), respectively. In the *∆feoAB ∆furA* strain, porphyrins increased approximately sevenfold under iron‐restricted conditions (0.153 nmol/mg of dried weight) compared to iron‐replete conditions (0.02 nmol/mg of dried weight) (Figure 3b).

These findings clearly show that accumulation of heme‐derived porphyrin occurs in the presence of heme when free iron is not available. They also demonstrate that *B. fragilis* is able to remove iron from heme releasing free‐protoporphyrin in a Fur‐independent manner, indicating the presence of a yet to be identified iron‐regulated heme dechelatase mechanism.

### 3.3 Iron inhibits the formation of the heme‐derived porphyrins

**Figure 4:**
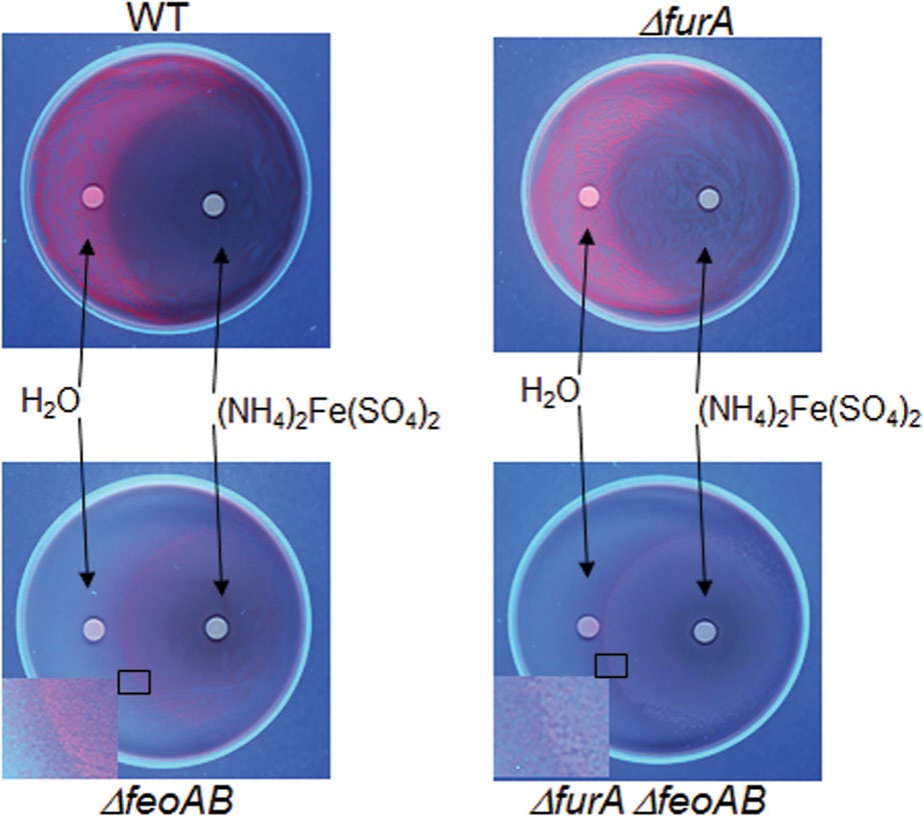
Growth of *Bacteroides fragilis* strains on brain heart infusion (BHIS) media containing 100 μg/ml hemin plus 1 mM bathophenanthroline disulfonic acid (BPS). Bacteria were spread on the surface of the plates and sterile disk filter papers were placed on top of the agar. 10 μl of sterile 0.5 M ammonium ferrous sulfate in double‐deionized H_2_O or 10 μl sterile double‐deionized H_2_O were added on respective disk filter papers as indicated in the panels. Bacteria were grown in an anaerobic chamber incubator at 37°C for 7 days. Plates were illuminated with a 365 nm UV‐long wave lamp (UVP; model UVLS‐28, Upland, CA) and pictures were taken with an Olympus Camedia C‐4000 digital camera. WT:* B. fragilis* 638R wild type. Strain designations are depicted for each panel. Bottom panel insets depict the bacterial growth edge area around the disk diffusion zone

To confirm that excess iron had an inhibitory effect on the production of heme‐derived porphyrin, *B. fragilis* strains were spread on BHIS agar supplemented with 100 μg/ml heme and 1 mM BPS. Then a filter paper disk was placed on top of the agar and 10 μl 0.5 M ammonium ferrous sulfate solution was added on top of the filter paper (Figure 4). Sterile water was used as control. The parent and ∆*furA* mutant strains were able to grow on the entire plates containing heme as the only source of exogenous iron. Formation of fluorescent heme‐derived porphyrin was observed further away from the iron diffusion zone around the disk filter impregnated with ammonium ferrous sulfate. In contrast, no fluorescence was observed around the disk diffusion area, indicating that excess of inorganic iron was able to inhibit formation of heme‐derived porphyrin (Figure 4, top panels). The ∆*feoAB* and ∆*feoAB* ∆*furA* double mutant strains only grew around the disk filter and not further away from the iron diffusion zone (Figure 4, bottom panels). Moreover, no fluorescence was observed for the *feoAB* or ∆*feoAB* ∆*furA* strains except on the edge of the lawn where iron was likely limiting (Figure 4, bottom panels inset). These findings show that FeoAB plays a central role in the acquisition of heme‐iron confirming that iron is removed from heme in the periplasmic space, and that formation of heme‐derived porphyrin is iron regulated in a Fur‐independent manner.

### 3.4 Reverse‐phase HPLC analysis of heme‐derived porphyrin from whole cell extracts

**Figure 5:**
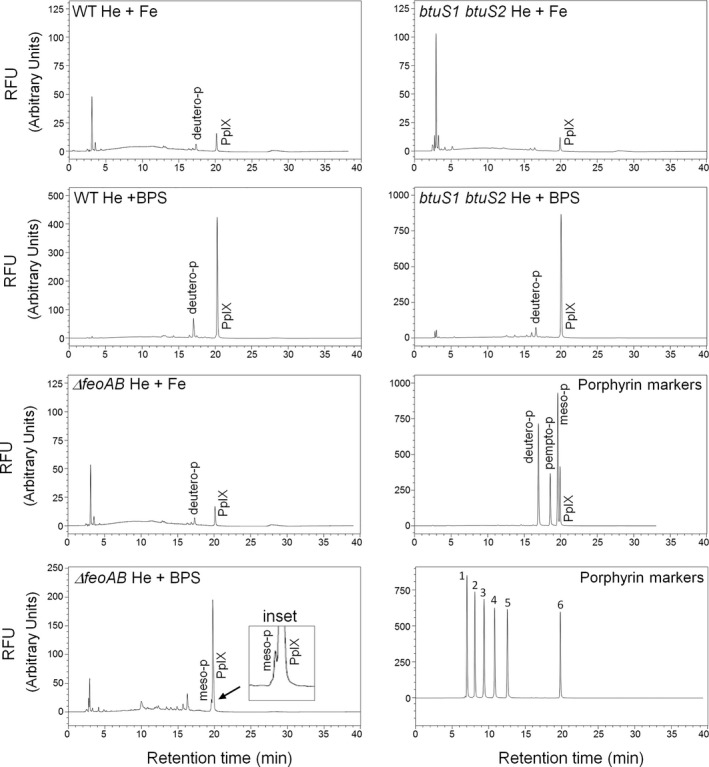
Chromatograms of free porphyrin acids analyzed by reverse‐phase high‐performance liquid chromatography. Total porphyrins were extracted in acid phase from dried whole cells of *Bacteroides fragilis* strains grown in brain heart infusion (BHIS) media containing 100 μg/ml heme (He) and supplemented with 100 μM ammonium ferrous sulfate (Fe) or 1 mM bathophenanthroline disulfonic acid (BPS). For growth of the *∆feoAB* strain under iron‐limiting conditions, 250 μM BPS was used. The inset graph is an enlargement showing a minor peak with retention time equivalent to mesoporphyrin IX. The procedures for porphyrin acid extracts, gradient separation conditions and detection settings are described in the material and methods section. Chromatographic porphyrin markers were used to identify peaks based on their retention times. deutero‐p: Deuteroporphyrin IX. pempto‐p: Pemptoporphyrin. meso‐p: Mesoporphyrin IX. PpIX: Protoporphyrin IX. 1: Uroporphyrin I (8 carboxyl porphyrin). 2: 7 carboxyl porphyrin. 3: 6 carboxyl porphyrin, 4: 5 carboxyl porphyrin. 5: Coproporphyrin I (4 carboxyl porphyrin). 6: Mesoporphyrin IX (2 carboxyl porphyrin). RFU: Relative fluorescence units

Analysis of porphyrin extracts of strains grown in the presence of 100 μg/ml heme plus ammonium ferrous sulfate or plus BPS showed major peaks virtually identical to the retention time of PpIX standard (Figure 5). The relative intensity of the PpIX peaks in cultures under inorganic iron restricted conditions were elevated approximately 10–20‐fold for the parent and ∆*feoAB* strains compared to the peak intensity under iron replete conditions. Other minor peaks were also observed in the chromatogram which overlapped well with the retention time observed for a deuteroporphyrin IX standard. Interestingly, a peak similar to the retention time of mesoporphyrin IX was also observed in ∆*feoAB* cultures grown under iron restricted conditions (Figure 5 inset). The specific organic structure and chemical composition of these porphyrins was not further determined. Nonetheless, these findings clearly show that *B. fragilis* possesses a dechelatase mechanism which is regulated by inorganic iron availability, and we also show that removal of iron from heme does not break the tetrapyrrole macrocycle structure hence releasing free PpIX. Moreover, these findings demonstrate that *B. fragilis* is able to modify the PpIX side‐chain. No detectable traces of porphyrins were found in uninoculated culture media (Supporting Information Figure S2).

### 3.5 Genetic analysis of CobN‐like chelatases in *B. fragilis*

A search in the genome of *B. fragilis* 638R strain revealed the presence of two putative operons (BF638R_2501‐2512 and BF638R_2714‐2721) containing CobN‐like chelatases belonging to the BtuS family; BF638R_2504 (BtuS1) and BF638R_2718 (BtuS2), respectively (Supporting Information Figure S3). The BtuS family of chelatases have been proposed as a third type of CobN‐related protein, distinct from the B12‐regulated cobalt chelatase CobN involved in the cobalamin synthesis, and the magnesium chelatase ChlH required for the bacteriochlorophyll biosynthesis (Rodionov et al., 2003). The BtuS family of proteins (pfam02514) contains a domain common to the CobN protein and to the magnesium protoporphyrin chelatase (https://www.ncbi.nlm.nih.gov/Structure/cdd/cddsrv.cgi?uid=pfam02514). Though BtuS is predicted to be involved in the salvage of metalloporphyrins rather than cobalamine biosynthesis (Rodionov et al., 2003), its role in anaerobic heme assimilation and utilization in *B. fragilis* has not been investigated. The BF638R_2714‐2721 operon contains homologs to the iron‐induced *hmu* locus for heme and hemoglobin utilization in *Porphyromonas gingivalis* (Lewis, Plata, Yu, Rosato, & Anaya, 2006; Simpson, Olczak, & Genco, 2000) suggesting that BtuS1 and BtuS2 could play a role in heme assimilation in *Bacteroides*. A phylogenetic analysis of BtuS homologs from *B. fragilis* with other CobN and CobN‐like homologs from bacteria and archaea phyla showed that BtuS1 and BtuS2 are branched in two distinct groups within the Bacteroidetes phylum (Supporting Information Figure S4). Most *Bacteroides* species contain a single homolog of *btuS* with the exception of nearly all *B. fragilis* strains which carry both the *btuS1* and the *btuS2* homologs. Interestingly, homologs of *btuS* gene were found to be absent in the genomes of *Bacteroides vulgatus* 20‐15 and 40G2‐33 strains (unpublished data) which are unable to grow on heme alone as the source of iron (Rocha & Krykunivsky, 2017). Moreover, transmembrane protein topology predictions using web‐based servers TMMOD: Hidden Markov Model for transmembrane protein topology (http://liao.cis.udel.edu/website/webscripts/frame.php?p=home) (Kahsay et al., 2005) and membrane topology and signal peptide analysis graphic visualization tools Protter (http://wlab.ethz.ch/protter/#) (Omasits, Ahrens, Müller, & Wollscheid, 2014) revealed that BtuS1 and BtuS2 are predicted to be exported with membrane attachment domains on the periplasmic side (Supporting Information Figure S5). These findings suggest that BtuS1 and BtuS2 might be involved in removing iron from heme in the periplasmic space. This correlates nicely with the findings shown above demonstrating that the inner membrane transporter FeoAB is required for the assimilation of iron released from heme outside of the cytoplasm. Therefore, *btuS1* and *btuS2* genes were chosen for further characterization.

### 3.6 Role of the CobN‐like proteins BtuS1 and BtuS2 during growth with heme as the sole source of iron

**Figure 6:**
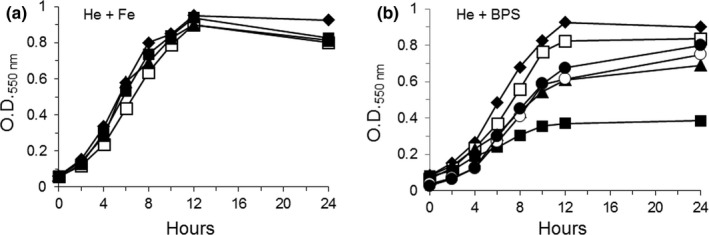
Growth of *Bacteroides** **fragilis* strains in defined medium containing 5 μg heme/ml (He). Media was supplemented with 100 μM ammonium ferrous sulfate (Fe) (a) or 400 μM bathophenanthroline disulfonic acid (BPS) (b) to obtain iron replete and iron‐limiting conditions respectively. ♦ *B. fragilis* 638R wild type; □ BER‐107 (*btuS1*); ▲ BER‐109 (*btuS2*); ■ BER‐111 (*btuS1 btuS2*); ○ BER‐114 (*btuS1 btuS2 btuS1* ^*+*^); ● BER‐115 (*btuS1 btuS2 btuS2* ^*+*^). The growth curves for BER‐114 and BER‐1115 strains are only shown in (b). Data presented are an average of two determinations in duplicate

To test the role of BtuS proteins in the utilization of heme‐iron, bacteria were grown in DM containing 5 μg/ml heme and supplemented with 100 μM ammonium ferrous sulfate or 500 μM BPS. In the presence of heme and inorganic iron replete conditions, no growth defect was observed for the *btuS1*,* btuS2* or *btuS1 btuS2* double mutant strains compared to the parent strain (Figure 6a). However, when bacteria were grown with heme alone as a sole source of iron, the *btuS1 btuS2* double mutant showed a growth defect compared to the parent and single mutant strains (Figure 6b). The *btuS1* single mutant did not show a significant growth defect while *btuS2* mutant showed a partial decrease in growth rate compared to parent strain. Growth of complemented strains was restored in part compared to the parent strain. The lack of complete complementation in the mutant strain may have been due to a polar mutation effect disrupting the expression of downstream genes which appear to be organized in a polycistronic operon. Moreover, genetic complementation carried out with single *btuS1* or *btuS2* genes in a multicopy plasmid may also have contributed to a pleiotropic effect. Nonetheless, this indicates that BtuS proteins are involved in acquisition of heme‐iron. However, to our surprise, the *btuS1 btuS2* double mutant strain did not abolish the ability of *B. fragilis* to remove iron from heme releasing free PpIX (Figure 5). In fact, the relative intensity of the PpIX peak in the *btuS1 btuS2* double mutant increased approximately twofold compared to the parent strain, as determined by reverse‐phase HPLC analysis. This suggests that BtuS1 and BtuS2 had no or little dechelatase enzymatic activity, but in contrast, their disruption increased formation of free PpIX. This is intriguing because formation of free PpIX in the *btuS1 btuS2* double mutant indicates that the iron released from heme does not appear to be efficiently assimilated to support growth as addition of exogenous inorganic iron can restore the growth defect phenotype.

### 3.7 BtuS modulates dechelatase and ferrochelatase activities in vitro

**Figure 7:**
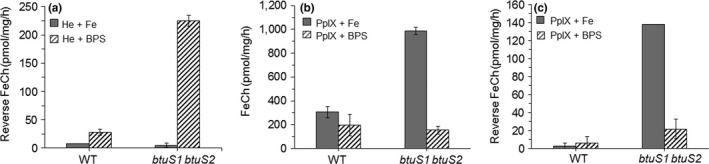
Ferrochelatase (FeCH) and reverse ferrochelatase activity assays in crude extracts of *Bacteroides fragilis* 638R (WT) and *btuS1 btuS2* double‐mutant strains. Bacteria were grown in brain heart infusion (BHIS) medium containing 10 μg heme/ml (He) or 10 μg protoporphyrin IX (PpIX)/ml. Media was supplemented with 100 μM ammonium ferrous sulfate (Fe) or 500 μM bathophenanthroline disulfonic acid (BPS). Bacteria were grown anaerobically at 37°C for 24–48 hr under iron replete conditions and for 72–96 hr under iron‐limiting conditions. Details of the forward ferrochelatase and reverse ferrochelatase reaction assay settings are described in the material and methods section

Though functional *btuS1* and *btuS2* genes are not required for *B. fragilis* to dechelate heme, they are involved in the regulation of the forward and reverse chelatase enzymatic activities measured in the cellular crude extracts. When *B. fragilis* was grown with heme under inorganic iron limited conditions, the reverse chelatase specific activity increased approximately eightfold in the *btuS1 btuS2* double mutant (225 pmol mg^−1^ hr^−1^) compared to the specific activity (28 pmol mg^−1^ hr^−1^) in the parent strain grown under iron restricted conditions (Figure 7a). In contrast, under iron replete conditions, the reverse chelatase specific activity in the parent strain (8 pmol mg^−1^ hr^−1^) did not significantly differ from the *btuS1 btuS2* double mutant (5 pmol mg^−1^ hr^−1^).

However, when bacteria were grown in the presence of PpIX, the reverse chelatase activity increased approximately 50‐fold in the *btuS1 btuS2* double mutant (138 pmol mg^−1^ hr^−1^) compared to the parent strain (2.8 pmol mg^−1^ hr^−1^) under iron replete conditions (Figure 7c). Under iron limitation, the reverse chelatase activity was elevated approximately 3.5‐fold in the *btuS1 btuS2* double mutant (21.4 pmol mg^−1^ hr^−1^) compared to the parent strain (6.1 pmol mg^−1^ hr^−1^) (Figure 7c). Moreover, the forward chelatase activity increased approximately threefold in the *btuS1 btuS2* double mutant (986 pmol mg^−1^ hr^−1^) compared to the parent strain (305 pmol mg^−1^ hr^−1^) under iron replete conditions (Figure 7b). In contrast, the forward chelatase activity did not change significantly in the parent strain (194.9 pmol mg^−1^ hr^−1^) compared to the *btuS1 btuS2* double mutant strain (153.9 pmol mg^−1^ hr^−1^) under iron limited conditions (Figure 7b).

Overall these findings demonstrate that the ability to dechelate heme is upregulated by iron limitation and that BtuS1 and BtuS2 proteins seem to have a negative regulatory effect. However, in the presence of PpIX, the negative regulatory effect is higher under iron replete conditions than under iron restricted conditions. This negative effect is also observed for the forward chelatase activity under iron replete conditions but in contrast to the reverse activity, the forward chelatase activity it is not regulated by iron restriction. Though the biochemical and genetic control mechanisms for the forward and reverse chelatase activities remain to be characterized, we show in this study that the BtuS1 and BtuS2 participate in the negative regulation of the forward and reverse chelatase activities of the heme metabolism in *B. fragilis*. In many bacteria, heme homeostasis is controlled at the initial steps of the biosynthetic pathway by HemX, a transmembrane anchored protein that regulates the abundance of glutamyl‐tRNA reductase, GtrR (Choby et al., 2018). In other bacteria such as the alpha‐proteobacteria, control of heme biosynthesis occurs through the direct interaction of the Irr regulator with the ferrochelatase to modulate gene expression under iron‐limiting conditions (Small, Puri, & O'Brian, 2009).

**Figure 8:**
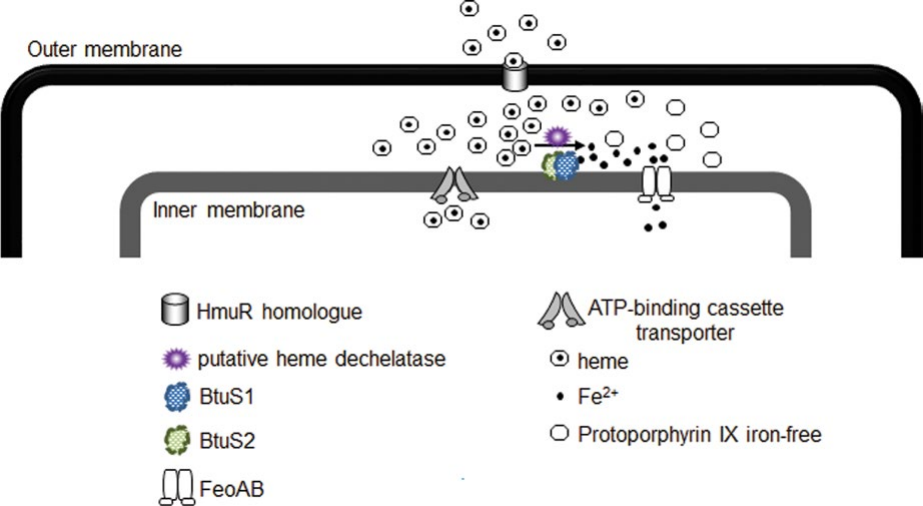
Schematic putative model of the involvement of the ferrous iron transporter FeoAB and the CobN‐like BtuS1 and BtuS2 proteins in the assimilation of heme‐iron in *Bacteroides fragilis*. Heme is transported into the periplasm through a HmuR homolog. Under iron‐limiting conditions, transported heme is dechelated releasing free‐protoporphyrin IX plus ferrous iron by an unidentified dechelatase enzyme in the periplasmic space. The iron released from heme requires the inner‐membrane ferrous iron transporter FeoAB system for assimilation into the cytoplasm. The putative periplasmic, inner‐membrane anchored BtuS1 and BtuS2 systems might participate in the regulation of the unidentified dechelatase enzyme

Therefore, we believe that the mechanism to extract iron from heme and make it available for cellular functions in *Bacteroide*s requires a group of proteins acting together, possibly forming a complex, which spans the periplasmic space. The BtuS1 and BtuS2 proteins described in this study are not the only components of this hypothetical protein machinery. At a minimum it also includes a dechelatase enzymatic activity, which is still undiscovered (Figure 8).

## 4 DISCUSSION

In this study, we show that the ability of *B. fragilis* to remove iron from heme releasing free PpIX is dependent on an unidentified dechelatase activity mechanism that is upregulated when bacteria are present in an iron‐limiting environment. Moreover, we demonstrate that utilization of iron removed from heme requires the inner membrane ferrous iron transporter system, FeoAB, indicating that the dechelatase activity occurs in the periplasmic space. It is well documented that members of the Bacteroidetes phylum such as *Porphyromonas* and *Prevotella* are able to release iron and form free PpIX from heme (Brazier, 1986; Fyrestam, Bjurshammar, Paulsson, Johannsen, & Östman, 2015; Fyrestam et al., 2017; Shah, Bonnett, Mateen, & Williams, 1979; Slots & Reynolds, 1982; Smalley & Olczak, 2017; Soukos et al., 2005). The removal of iron from heme in *Prevotella melaninogenica* (formerly black‐pigmented *Bacteroides melaninogenicus*) occurs by the action of an unidentified demetallase, which was elegantly demonstrated by Shah et al. (1979). Other studies have suggested that the ability of *P. gingivalis* to remove iron from heme could be due to the reverse activity of its ferrochelatase HemH (Olczak et al., 2005). It has also been suggested that the *P. gingivalis* outer membrane cobalt chelatase CbiK‐like protein IhtB may remove iron from heme extracellularly (Dashper et al., 2000). However, the lack of ferrochelatase HemH in *B. fragilis* (Rocha & Smith, 2010) and the findings that disruption of the cobalt chelatase *ckiK* gene (BF638R‐2502) did not affect utilization of heme‐iron (unpublished data) indicate that the dechelatase mechanism in *B. fragilis* differs from the ones proposed for the related organism *P. gingivalis*. The disruption of the BF638R_2327 gene, a homolog to *yfeX* belonging to the DYP‐peroxidase family of proteins which is implicated in heme dechelation in *E. coli* (Létoffé, Heuck, Delepelaire, Lange, & Wandersman, 2009) did not affect removal of iron from heme in *B. fragilis* (unpublished data). Moreover, the deferrochelatase/peroxidase *efeB* in *E. coli* (Létoffé et al., 2009), has no homolog in *B. fragilis* (data not shown). These findings suggest that *Bacteroides* spp. have developed a novel mechanism(s) to utilize heme‐iron.

It seems that there is dual coordinated regulation of heme‐iron utilization in *B. fragilis*. Firstly, the FeoAB transporter, necessary for the utilization of ferrous iron released from heme, is regulated by iron in a Fur‐dependent manner (Rocha & Krykunivsky, 2017). Secondly, the removal of iron from heme by dechelatase activity, modulated by BtuS1 and BtuS2 proteins, is regulated by iron availability in a Fur‐independent manner. The transport of ferrous iron in intestinal anaerobes is still poorly understood. In aerobic and facultative anaerobic bacteria the Feo system is generally composed of the small cytoplasmic protein FeoA and the large transmembrane protein FeoB. This stands in contrast to many upper and lower GI tract anaerobes such as *Porphyromonas*,* Bacteroides Eubacterium*,* Ruminococcus,* and *Clostridium* species which contain the *feoA* and *feoB* genes encoded in a single fused polypeptide (Dashper et al., 2005; Rocha & Smith, 2010; Veeranagouda et al., 2014). The translated interpeptide amino acid residues between the FeoA and FeoB domains are highly conserved among the Bacteroidetes, and among the Firmicutes and Actinobacteria phyla of intestinal colonizers (Rocha & Smith, 2010). As far we are aware, fused FeoAB peptides are not found in nongastrointestinal organisms suggesting its unique importance for the physiology of intestinal anaerobic bacteria (Rocha & Smith, 2010). We have previously shown that the lack of FeoAB impaired *B. fragilis* to form abscesses in a mouse model of infection (Veeranagouda et al., 2014). However, in light of this study, this growth deficiency is likely to have been due to the *∆feoAB* strain inability to acquire both heme‐iron and inorganic iron from host tissues.

It is not clear how BtuS1 and BtuS2 affect the regulation of both the chelatase and dechelatase enzymatic activities as demonstrated in the in vitro assays using whole cell extracts. We assume that the effect of CobN‐like BtuS family of proteins on the ferrochelatase and dechelatase activities may provide advantageous benefits for *B. fragilis* pathophysiology and commensal intestinal colonization. At least with regard to the dechelatase activity, it appears to be located in the periplasmic space. At this point of investigation, it is unclear how BtuS1 and BtuS2 interact with and affect dechelatase activity, and the physiological significance of their regulatory effect. However, the fact that the iron released from heme in the periplasm seems to be less efficiently assimilated to support growth of the *btuS1 btuS2* double‐mutant, it points out a potential role for the BtuS1 and BtuS2 system in facilitating its utilization. It may be energetically cost effective to remove iron from heme or modify the porphyrin side‐chain in the periplasmic space rather than transporting porphyrins across cellular membranes for export. It is possible that this mechanism might facilitate rapid exchange of porphyrins and side chain‐modified porphyrins among intestinal bacteria, especially in view of the fact that *B. fragilis* can incorporate side chain‐modified heme into cytochrome *b*‐type (for review see Rocha & Smith, 2010).

Recent studies have demonstrated that the oral pathogens *P. gingivalis*,* Aggregatibacter actinomycetemcomitans*,* Prevotella intermedia*,* Prevotella nigrescens*, and *P. melaninogenica* produce endogenous porphyrins when grown on media containing animal blood (Fyrestam et al., 2015; Soukos et al., 2005). The *Porphyromonas* and *Prevotella* porphyrins were mostly PpIX, coproporphyrinogen I, and coproporphyrinogen III (Fyrestam et al., 2015, 2017; Soukos et al., 2005). However, no side chain‐modified porphyrin such as mesoporphyrin IX or deuteroporphyrin IX were reported to be formed by these oral bacteria (Fyrestam et al., 2015, 2017; Soukos et al., 2005). Therefore, the presence of PpIX, mesoporphyrin IX and deuteroporphyrin IX found in *B. fragilis* suggests that this organism contributes to the appearance of heme‐derived porphyrins and side chain‐modified porphyrins in extraintestinal infections as well as in the intestinal tract. This is in agreement with previous studies demonstrating that intestinal anaerobic bacteria are exclusively responsible for the conversion of heme to PpIX lacking iron, and modifications of the vinyl side chains of heme and PpIX to their respective deutero, meso, and pempto side chain forms (Beukeveld et al., 1987; Young et al., 1989, 1990). There is a wide range of variations in the amount and type of heme‐derived porphyrins among individuals, but the types of heme‐derived porphyrins seem to be consistent within a given individual (Young et al., 1990). Though fecal flora metabolism and redox potential may account for the differences in the amount and types of heme‐derived porphyrins (Young et al., 1990), we demonstrate in this study that intestinal iron limitations may also contribute to the fluctuations in the amount of heme‐derived porphyrins formed by intestinal anaerobic bacteria.

Though heme has been shown to be inhibitory to many Gram‐positive and Gram‐negative bacteria in vitro (Nitzan, Wexler, & Finegold, 1994), dietary heme has a robust effect in enhancing the abundance of Bacteroidetes relative to the Firmicutes population in mouse intestinal colonization models (Ijssennagger et al., 2012, 2015). In addition to this effect on bacterial colonization, heme‐induced hyperproliferation and hyperplasia in the mouse colon only occurs in the presence of the gut microbiota (Ijssennagger et al., 2015). The specific bacterial species involved in such mechanisms have not been identified, and whether there is a link between bacterial action on modifications of heme structure and heme‐derived porphyrins in enhancing or weakening heme mucosa toxicity remains to be determined. In this study we identify *B. fragilis* as being one of these bacteria able to act on and modify labile heme molecules in vitro. Because formation of heme‐derived porphyrin seems to be regulated by free iron availability, we think that heme‐derived porphyrins might be relevant for *B. fragilis* pathophysiology in extraintestinal infections where iron is limited by the host. This assumption is based on the fact that protoporphyrin IX acts as a competitive inhibitor of the proinflammatory activity of labile heme in macrophages (Figueiredo et al., 2007; Soares & Bozza, 2016). Moreover, we speculate that by modifying or scavenging labile heme in the site of infection, *B. fragilis* may alter the signaling effect of labile heme on recruitment of neutrophil and oxidative burst (Figueiredo et al., 2007; Soares & Bozza, 2016). Further investigation on heme‐iron utilization systems and regulation will advance our understanding on the role heme plays in *Bacteroides* pathophysiology.

## CONFLICT OF INTEREST

The authors have no conflict of interest to declare.

## Supporting information


** **
