# Supplementary material for: Bacteroides fragilis requires the ferrous‐iron transporter FeoAB and the CobN‐like proteins BtuS1 and BtuS2 for assimilation of iron released from heme
Source: Microbiologyopen. 2018 Jun 21;8(4):e00669. doi: 10.1002/mbo3.669 (PMC6460266; doi:10.1002/mbo3.669)
Supplement: Supplementary file 1 [file MBO3-8-e00669-s001.pdf]

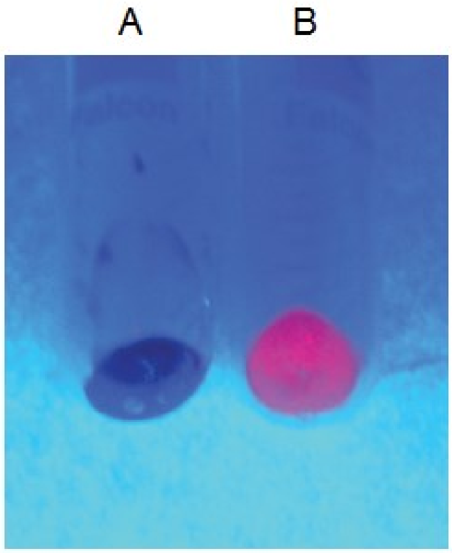

Supplemental Figure S1. *B. fragilis* 638R culture pellets. Bacteria were grown in BHIS containing 100 μg heme/ml and supplemented with 100 μM ammonium ferrous sulfate (A) or 1 mM bathophenanthroline disulfonic acid (B). After 48h incubation at 37° C anaerobically, bacterial cultures were harvested and the pellets were illuminated with a 365 nm UV-long wave lamp.

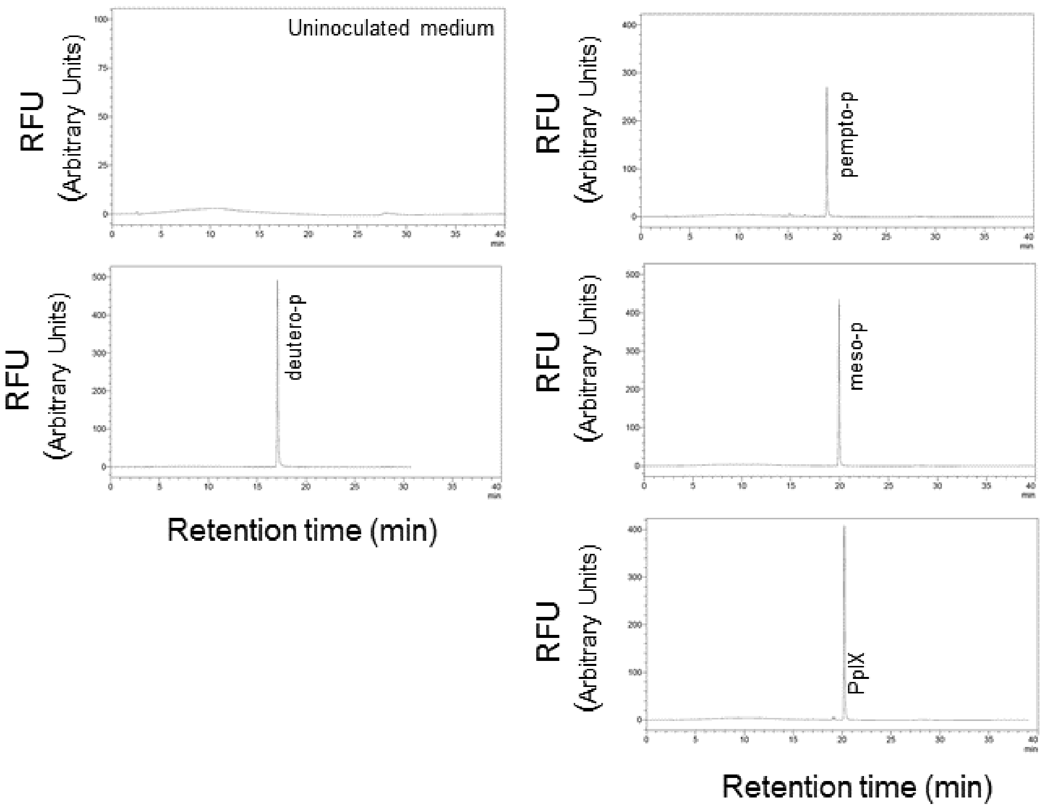

Supplemental Figure S2. Analyses of porphyrins extracted from uninoculated BHIS culture media containing 100 µg heme/ml. The procedures for porphyrin acid extracts, gradient separation conditions and detection settings are described in the material and methods section. Chromatographic porphyrin markers were used for peaks identification based on their retention times. deutero-p: deuteroporphyrin IX. pempto-p: pemptoporphyrin. meso-p: mesoporphyrin IX. PpIX: protoporphyrin IX.

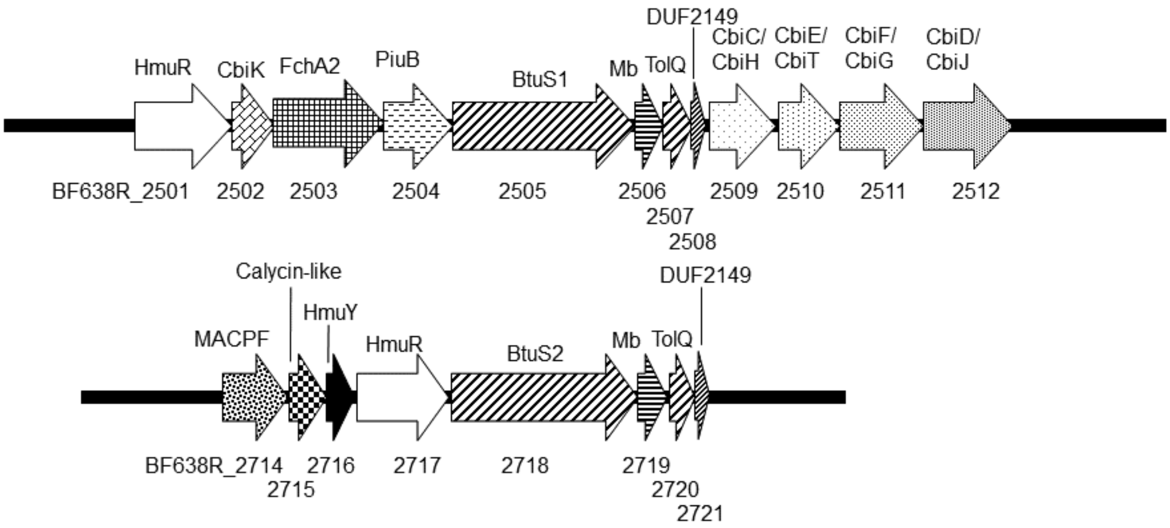

Supplemental Figure S3. Schematic representation of the *B. fragilis* 638R_2501-2512 and 2714-2721 loci region. Each tag locus is depicted below respective putative ORF symbolized by an arrow. Arrows filled with same shape pattern represent same functional annotation group assigned to respective locus tag. The name of the predicted translational product is depicted above each gene region respectively. Mb: putative membrane protein. HmuYR are homologues to the heme-binding protein HmuY and the TonB-dependent hemoglobin-heme receptor HmuR *in P. gingivalis* (Simpson et al., 2000). CbiK: anaerobic cobalt chelatase. FchA2: a homologue to *E. coli* ferrichrome transporter FhuA (Rocha and Krykunivsky, 2017). MACPF: membrane attack complex/perfori with antimicrobial protein secreted by *B. fragilis* (Chatzidaki-Livanis et al., 2014). PiuB: iron-regulated membrane protein (COG3182). Calcylin-like: Protein containing Calycin-like beta-barrel domain homologue to *E. coli* YodA, a metal-binding lipocalin-like protein (David et al., 2003). Mb: putative membrane transporter. TolQ: membrane transport protein (COG0811). DUF2149: Uncharacterized conserved protein. CbiC/CbiH: This family of proteins contain a domain common to the precorrin-8x methylmutase (CbiC) and precorrin_3B_C17_methytransferase (CbiH). CbiE/CbiT: putative bifunctional precorrin-6Y-methylase and precorrin-6Y C5, 15-methytransferase. CbiF/CbiG: putative bifunctional cobalamin biosynthesis protein percorrin-4 C11methyltrasferase. CbiD/CbiJ: putative bifunctional cobalt-precorrin-6A synthase and precorrin 6x reductase for cobalamin biosynthesis.

References:

Chatzidaki-Livanis, M., Coyne, M. J., & Comstock, L. E. (2014). An antimicrobial protein of the gut symbiont Bacteroides fragilis with a MACPF domain of host immune proteins. Molecular Microbiology, 94, 1361– 1374. https://doi.org/10.1111/mmi.12839.

David, G., Blondeau, K., Schiltz, M., Penel, S., & Lewit-Bentley, A. (2003). YodA from Escherichia coli is a metal-binding, lipocalin-like protein. Journal of Biological Chemistry, 278, 43728–43735. https://doi. org/10.1074/jbc.M304484200.

Rocha, E. R., & Smith, C. J. (2010). Heme and iron metabolism in Bacteroides. In S. C. Andrews, & P. Cornelis (Eds.), Iron uptake and homeostasis in microorganisms, chapter 9 (pp. 153–165). Norwich, UK: Caister Academic Press.

Simpson, W., Olczak, T., Genco, C. A. (2000). Characterization and expression of HmuR, a TonB-dependent hemoglobin receptor of *Porphyromonas gingivalis*. J Bacteriol. 182:5737-5748. PMID: 11004172.

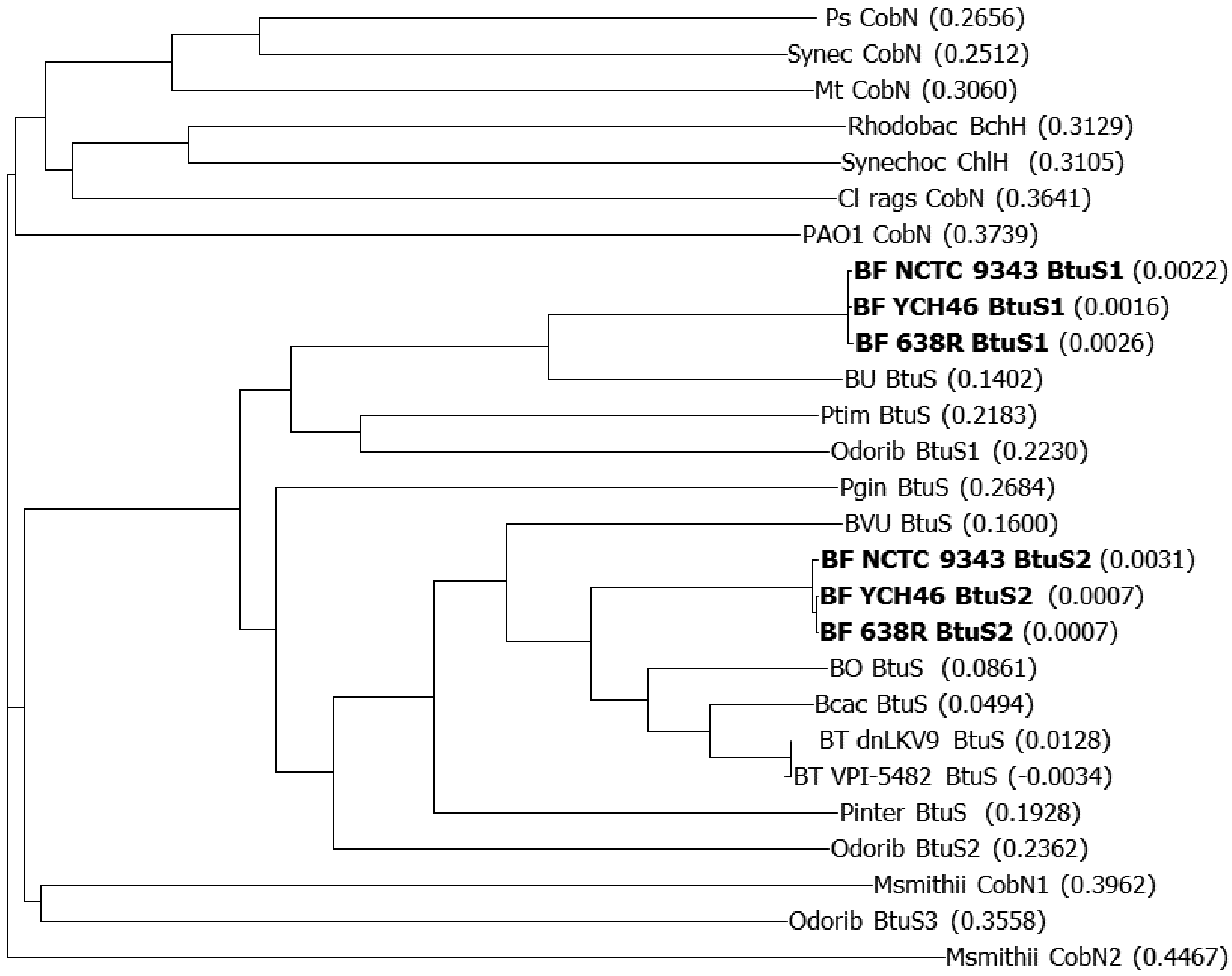

Supplemental Figure S4. Phylogenetic relationship of 27 cobalt chelatatase subunit CobN and CobN-like BtuS chelatase protein homologues representatives from Bacteroidetes, Actinobacteria, Cyanobacteria, Euryachaeota, Firmicutes, Alphaproteobacteria and Gammaproteobacteria. The unrooted phylogenetic tree was constructed from multiple amino acid sequences alignment based on ClustalW algorithm in the AlignX program of Vector NTI 11.5.4. The Neighbor Joining method of Saitou and Nei (1987) was used to calculate the distances between all pairs and the calculated relative distance values are depicted in parenthesis following each tree entry name. GenBank accession numbers and abbreviations are as follow: Bcac: *Bacteroides caccae* ATCC 43185 (EDM22024). BF: *Bacteroides fragilis* 638R BtuS1 (CBW23013) and BtuS2 (CBW23214), BF NCTC 9343 BtuS1 (CAH08243) and BtuS2 (CAH08405), BF YCH46 ButS1 (BAD49263) and BtuS2 (BAD49439). Cl rags: *Clostridium ragsdalei* P11 CobN (OBR96985). BO: *Bacteroides ovatus* ATCC 8483 BtuS (EDO10378). BT dnLVK9: *Bacteroides thetaiotaomicron* dnLKV9 BtuS (EOS02610).BT VPI-5482: *Bacteroides thetaiotaomicron* VPI-5482 BtuS (AAO75601). BU: *Bacteroides uniformis* ATCC 8492 BtuS (EDO53638). BVU: *Bacteroides vulgatus* ATCC 8482 BtuS (ABR39855). Msmithii: *Methanobrevibacter smithii* ATCC 35061 CobN1 (ABQ87920) and CobN2 (ABQ87322). Mt: *Mycobacterium tuberculosis* H37Rv CobN (AIR14808). Odorib: *Odoribacter splanchnicus* DSM 20712 BtuS1 (ADY33428), BtuS2 (ADY32460), and BtuS3 (ADY33461). Pgin: *Porphyromonas gingivalis* W83 CobN (HmuS) (AAQ66589). Pinter: *Prevotella intermedia* ATCC 25611 BtuS (APW33271). Ptim: *Parabacteroides timonensis* BtuS (WP_075555722). PAO1: *Pseudomonas aeruginosa* PAO1 CobN (NP_250613). Ps: *Pseudomonas* sp. ATCC 13869 (AGI22494). Rhodobac: *Rhodobacter capsulatus* R121 BchH (ETD78925). Synec: *Synechocystis* sp. PCC 6803 CobN (WP_010872023) and ChlH (BAK49212).

References:

Saitou, N., & Nei, M. (1987). The neighbor-joining method: A new method for reconstructing phylogenetic trees. Molecular Biology and Evolution, 4, 406–425.

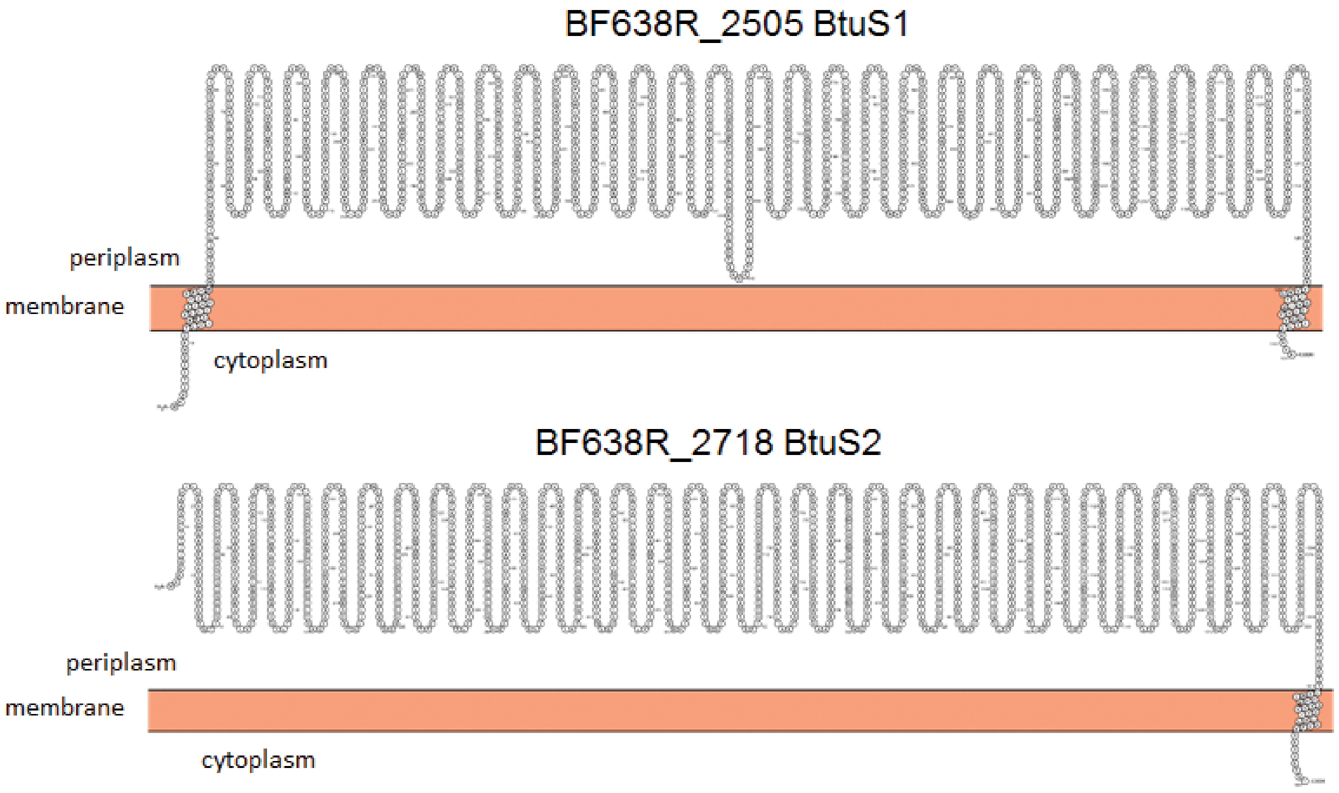

Supplemental Figure S5. Topology illustration of the predicted membrane anchored BtuS1 and BtuS2 proteins using putative amino acid sequences from *B. fragilis* 638R_2505 and 2718 gene loci respectively. Proteins were visualized using Protter v.1 (http://wlab.ethz.ch/protter/#).
